# Neurobiological mechanisms of psychosis in epilepsy: Findings from neuroimaging studies

**DOI:** 10.3389/fpsyt.2022.1079295

**Published:** 2022-11-23

**Authors:** Daichi Sone

**Affiliations:** Department of Psychiatry, Jikei University School of Medicine, Tokyo, Japan

**Keywords:** psychosis, epilepsy, comorbidity, functional neuroimaging, structural neuroimaging

## Abstract

Despite the high prevalence and clinical importance of comorbid psychosis in epilepsy, its neurobiological mechanisms remain understudied. This narrative mini-review aims to provide an overview of recent updates in *in vivo* neuroimaging studies on psychosis in epilepsy, including structural and diffusion magnetic resonance imaging (MRI) and functional and molecular imaging, and to discuss future directions in this field. While the conventional morphological analysis of structural MRI has provided relatively inconsistent results, advanced methods, including brain network analysis, hippocampal subregion volumetry, and machine learning models, have recently provided novel findings. Diffusion MRI, for example, has revealed a reduction in white matter integrity mainly in the frontal and temporal lobes, as well as a disruption of brain white matter networks. Functional neuroimaging, such as perfusion single-photon emission computed tomography (SPECT) or fluorodeoxyglucose positron emission tomography (FDG-PET), often identifies hyperactivity in various brain regions. The current limitations of these more recent studies may include small and sometimes heterogeneous samples, insufficient control groups, the effects of psychoactive drugs, and the lack of longitudinal analysis. Further investigations are required to establish novel treatments and identify clinical diagnostic or disease-monitoring biomarkers in psychosis in epilepsy.

## Introduction

It is known that patients with epilepsy suffer from psychiatric comorbidities with a 35% life-time prevalence (1). This occurrence is more frequent than in the general population (1), and can greatly affect quality of life of patients and their caregivers (2, 3). In addition, psychiatric symptoms sometimes occur prior to the onset of seizures and correlate with seizure outcomes (4); currently, a bidirectional relationship between epilepsy and psychiatric comorbidities is assumed. Both psychiatric disorders and epilepsy occur in the brain, and it is therefore possible that epilepsy-related changes in the brain may be associated with the development and manifestation of psychiatric symptoms. Although past studies have focused on endocrinology, neurotransmitters, brain structures, and immunology (4), the neurobiological mechanisms of psychiatric comorbidity in epilepsy remain to be elucidated.

Psychosis is a serious psychiatric condition characterized by hallucinations, delusions, and bizarre or disorganized behaviors (5). The odds ratio for psychosis in people with epilepsy is 7.8 times higher than in the general population (6), and historically, psychosis has received attention in terms of forced normalization or alternative psychosis, i.e., psychosis associated with a reduction in epileptiform discharges or seizures (5, 7). Temporal lobe epilepsy (TLE) has a greater prevalence (up to 20%) of psychosis, particularly postictal psychosis (8), while the remaining >80% of patients do not develop psychosis. Considering these epidemiological studies, it is reasonable to conclude that some neural mechanisms in epilepsy and seizure, especially the limbic circuit in TLE, may potentially be involved in the development of psychosis. Such bidirectional neurobiological mechanisms should also be supported by the evidence of beneficial effect of electroconvulsive therapy (ECT) on psychotic disorders (9). However, the neural basis of psychosis in epilepsy remains unknown and requires more investigation, particularly regarding what mechanisms underlie psychosis in epilepsy and whether they are different from those in the general population (e.g., schizophrenia).

Neuroimaging is a powerful tool that can investigate human brains non-invasively, and it has frequently been applied to investigations on various neuropsychiatric disorders (10). In fact, several past neuroimaging studies have reported various and varied findings in psychosis in epilepsy, with diverse and sometimes inconsistent results (11). More recently, advanced neuroimaging methods, such as brain network analysis, machine learning, and hippocampal subfields, have been applied and have provided novel findings.

The aim of this narrative review is to provide an overview of recent updates in *in vivo* neuroimaging studies on psychosis in epilepsy, including structural and diffusion magnetic resonance imaging (MRI), and functional and molecular imaging, and to discuss future directions in this field. In addition to neuroscientific progress, a better understanding of the neurobiological aspects of psychosis in epilepsy may potentially lead to novel treatments as well as to the identification of clinical diagnostic or disease-monitoring biomarkers. A search of literature was conducted in PubMed database on October 1, 2022 using “psychosis,” “epilepsy,” “MRI,” “PET,” and/or “SPECT” as key words, and relevant studies were manually selected, although no rigorous systematic selection criteria was adopted for this narrative review.

## Structural magnetic resonance imaging of psychosis in epilepsy

Earlier studies on brain morphological changes in psychosis in epilepsy focused primarily on mesial temporal lobe structures, such as the hippocampus and amygdala, using manual tracing methods. The hippocampal and amygdala findings of psychosis in epilepsy in these studies were diverse, including bilateral volume loss of the hippocampus and amygdala (12), no difference in total hippocampal volumes (13), left hippocampal volume reduction (14), widespread gray matter volume reduction (15), and bilateral amygdala enlargement (16).

**FIGURE 1:**
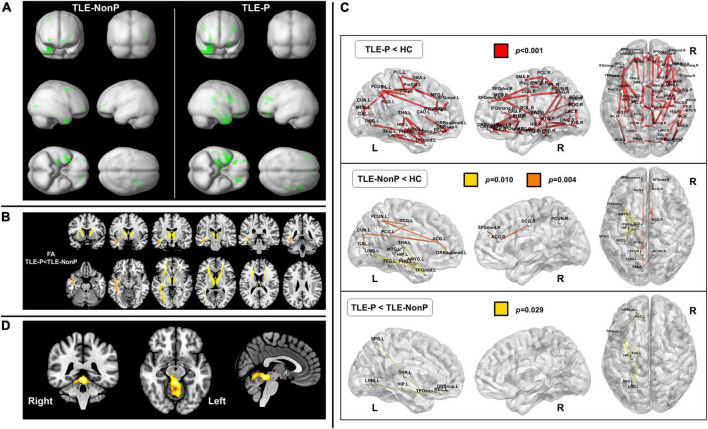
Neuroimaging findings in psychosis in epilepsy. **(A)** Gray matter reduction in temporal lobe epilepsy (TLE) with and without psychosis (TLE-P and TLE-NonP) compared with healthy controls, derived from structural magnetic resonance imaging (MRI). Both TLE-P and TLE-NonP showed significant volume reduction in the ipsilateral temporal and other brain areas, but there was no significant direct difference between them [Cited from Sone et al. (25)]. **(B)** Significant reduction in fractional anisotropy (FA) within the white matter tracts in TLE-P, derived from diffusion MRI [Cited from Sone et al. (40)]. **(C)** Disrupted brain white matter networks in TLE-P and TLE-NonP, derived from diffusion MRI [Cited from Sone et al. (40)]. **(D)** Hypermetabolic areas in TLE-P compared to TLE-NonP, derived from fluorodeoxyglucose positron emission tomography (FDG-PET) [Cited from Sone et al. (48)].

Along with the development of automated whole brain MRI analysis, such as voxel-based morphometry (17) or surface-based morphometry (18), trends in structural neuroimaging studies on psychosis in epilepsy have also changed. Since 2004, several automated brain morphometric studies have reported a variety of results in psychosis in epilepsy, such as bilaterally widespread gray and white matter reduction (19), increased and decreased cortical thickness (20), cortical thinning in the inferior frontal gyrus (21), gray matter reduction in the left parietal lobe (22), and no significant effect of psychosis on brain morphological changes (23–25) (Figure 1A).

While automated brain morphometry is suitable for whole-brain analysis, and is fully reproducible and more efficient in terms of time and effort, manual tracing is less error-prone and more accurate when rigorously applied. However, in the literature to date, neither automated nor manual methodologies have succeeded in providing consistent results, and thus simple brain morphology may be insufficient to reveal the neural mechanisms of psychosis in epilepsy.

Recently, several advanced analytical methods for neuroimaging have emerged and are expected to provide further evidence on epilepsy. These include brain network analysis (26), machine learning (27), and hippocampal subfield analysis (28). Epilepsy-related alterations are usually distributed across multiple brain regions beyond a single focus, e.g., the hippocampus in TLE, and thus epilepsy is currently considered a network-level disorder (26). As this concept of network neuroscience could be applied to psychiatric disorders (29), psychosis in epilepsy may also benefit from brain network analysis. In a previous study using graph theoretical analysis based on gray matter anatomical covariance network (25), TLE with psychosis showed a significantly higher characteristic path length and transitivity, and lower global efficiency, as well as reduced resilience to attacks, compared to TLE without psychosis. In addition, a recent study identified increased cortical thickness in psychosis in epilepsy, primarily in the cognitive control network and default mode network areas (30).

The hippocampal formation consists of major subfields, i.e., the cornu ammonis, as well as other subregions such as the dentate gyrus and subiculum. These hippocampal subregions have different functions and neural pathways (31). Functional organization along the long axis of the hippocampus is also attracting attention (32). Thus, hippocampal subfield and/or subregion analysis is expected to provide further knowledge about epilepsy, particularly TLE, beyond what can be learned by analyzing the hippocampus as a single region. In psychosis in epilepsy, bilateral posterior hippocampal atrophy has been identified, while the head and body of the hippocampi were not affected (33). According to another recent study, hippocampal subfield volumes in psychosis in epilepsy were not significantly different from those in epilepsy without psychosis, while the hippocampal fissure was significantly enlarged in psychosis in epilepsy (34).

Machine learning analysis has the advantage over conventional methods of accurate, automated, and fast pattern learning and is expected to lead to optimal algorithms for clinical neuropsychiatry and epilepsy (27). One of the emerging trends in machine learning for neuropsychiatry is the brain-age prediction framework, which estimates the age of each individual’s brain image using a machine learning regression model (35, 36). Given the close relationship between aging and neuropsychiatric disorders, brain-age prediction may contribute as a clinically useful, individualized biomarker. In TLE with interictal psychosis, brain age has been found to be higher than chronological age by 10.6 years, and the gap was significantly higher than that in TLE without psychosis (+5.3 years) (37). It is possible that accelerated aging and a brain that appears older may be suggested as potential biomechanisms of psychosis in epilepsy. Thus, several advanced methodologies have been applied to psychosis in epilepsy, and further work may expand our knowledge.

## Diffusion magnetic resonance imaging of psychosis in epilepsy

Diffusion tensor imaging (DTI) is sensitive to water diffusion features, and diffusion anisotropy can be used as a marker for brain white matter tract integrity (38). In TLE with interictal psychosis, one study identified significantly lower fractional anisotropy in the bilateral frontal and temporal white matter regions, compared with TLE without psychosis, using manual region-of-interest (ROI) delineation (39). More recently, another study applied whole-brain tract-based analysis of DTI and reported a more widespread reduction in white matter integrity in TLE with interictal psychosis, and in particular, the anterior thalamic radiation, inferior fronto-occipital fasciculus, and inferior longitudinal fasciculus were damaged compared with TLE without psychosis (40) (Figure 1B), which seems consistent with the previous study (39). In addition, white matter structural network analysis revealed significantly reduced global and local efficiency as well as network abnormalities involving the left limbic and prefrontal areas in TLE with interictal psychosis (40) (Figure 1C). While DTI studies on psychosis in epilepsy remain scarce, their results are expected to be more consistent than those of structural MRI studies.

## Functional neuroimaging of psychosis in epilepsy

Most functional neuroimaging studies on psychosis in epilepsy have utilized single-photon emission computed tomography (SPECT) to measure cerebral blood flow. Interictally, the reported findings range from hypoperfusion in the left superior temporal gyrus (41) to no significant differences (42). During the psychotic period, hyperperfusion findings have been reported in the lateral temporal lobe (43), fronto-temporal lobes (44, 45), and right temporal lobe (46). ^18^F-fluorodeoxyglucose positron emission tomography (FDG-PET) is also widely used in clinical practice in cases of epilepsy (47), but FDG-PET studies on psychosis in epilepsy are more limited compared with perfusion SPECT studies. A recent study found brain glucose hypermetabolism in the upper cerebellum, superior cerebellar peduncle, and midbrain (48) (Figure 1D), which might be associated with cerebellar involvement in cognition and emotion (49, 50). Thus, most functional neuroimaging studies have suggested brain hyperactivity in psychosis in epilepsy, while the reported abnormal areas are diverse. Apart from nuclear imaging, reduced N-acetyl aspartate (NAA) within the hippocampi has been reported in psychosis in epilepsy (12). On the other hand, the lack of functional MRI studies may be notable, given the abundant applications to psychiatric disorders (51). While FDG-PET and perfusion SPECT are widely used in clinical practice for epilepsy (52), the clinical use of functional MRI is still limited, which might be a cause of the discrepancy. At any rate, considering the advantage for non-invasive evaluation of dynamic brain activity, future studies on functional MRI findings in psychosis of epilepsy should be desirable.

## Future directions

Compared with the substantial neuroimaging evidence regarding pure psychiatric disorders and common types of epilepsies, psychosis in epilepsy is distinctly understudied. Additionally, the cohorts in these studies are small (up to *N* = 30) and sometimes heterogeneous in terms of type of epilepsy and type of psychosis, e.g., mixed cohorts with postictal and interictal psychoses. Furthermore, most studies do not include a control cohort of psychosis without epilepsy, e.g., schizophrenia. To reveal the neurobiology of psychosis in epilepsy, it is desirable to compare psychosis in epilepsy with epilepsy without psychosis, psychosis without epilepsy, and healthy subjects. Moreover, most patients in these studies take antipsychotic medications, and thus the effects of drugs must be taken into consideration. Further longitudinal investigation on drug-naïve cases from the early stage of psychosis may address such problems and could lead to more useful imaging biomarkers for prediction and disease-monitoring. Finally, further evidence may be provided by other advanced imaging techniques that have not yet been used to study psychosis in epilepsy, such as functional MRI, multi-shell diffusion MRI, or specific PET tracers.

## Conclusion

There have been efforts to reveal the neurobiological mechanisms of psychosis in epilepsy using structural and functional neuroimaging. Though results from structural MRI are relatively inconsistent, advanced imaging techniques are now being applied and provide further knowledge on this condition. Psychosis in epilepsy remains understudied, however, and additional research is required in order to address the current issues.

## Author contributions

The author confirms being the sole contributor of this work and has approved it for publication.
